# The role of pruriceptors in enhancing sensitivity to pruritogens in a murine chronic compression model of dorsal root ganglion

**DOI:** 10.1186/s13041-021-00730-9

**Published:** 2021-01-19

**Authors:** Tao Wang, Jin Tao, Yehong Fang, Chao Ma

**Affiliations:** 1 Institute of Basic Medical Sciences, Neuroscience Center, Chinese Academy of Medical Sciences, Peking Union Medical College, Beijing, 100005 China; 2 Department of Human Anatomy, Histology and Embryology, School of Basic Medicine, Peking Union Medical College, Beijing, 100005 China; 3 Joint Laboratory of Anesthesia and Pain, Peking Union Medical College, Beijing, 100730 China

**Keywords:** DRG compression, Pruriceptor, Itch, Hyperkinesis, Behavior

## Abstract

Chronic pruritus is a symptom that commonly observed in neurological diseases. It has been hypothesized that the chronic pruritus may result from sensitization of itch-signaling pathways but the mechanisms remain obscure. In this study, we established a mouse model of chronic compression of dorsal root ganglion (CCD) and injected various pruritogenic and algogenic agents intradermally to the calf skin ipsilateral to the compressed dorsal root ganglion (DRG). Compared to the naïve mice, a significant increase in itch-related behaviors was observed in the CCD mice after the injection of pruritogens including histamine and BAM8-22, but not after the injection of capsaicin, although all the above agents evoked enhanced pain-related behaviors toward the injected site. In addition, we investigated if pruritogen-evoked activities of DRG neurons were enhanced in this model. In vivo calcium imaging revealed that compressed DRG neurons exhibited enhanced responses to histamine and BAM8-22. Immunoflorescent staining also showed that the histamine receptor H1 and the capsaicin receptor TRPV1 were significantly upregulated in DRG neurons. Our findings indicated that the sensitization of primary pruriceptive neurons may underlie the enhanced itch sensation after chronic compression of DRG in the mice, and may play a role in chronic pruritus in neurological diseases.

## Introduction

Itching (pruritus) has been defined as an “unpleasant skin sensation that elicits the desire or reflex to scratch’’ [1]. Primary sensory neurons in dorsal root ganglia (DRG) play an important role in generating itch by detecting pruritogenic stimuli via their peripheral axons in the skin and sending the signals to the spinal cord through their central axons [2]. Although itch and pain are both somatosensory sensations achieved by activating sensory nerves, they can be differentiated by psychophysiological and molecular characteristics [3]. Compression of the cervical spinal cord or the spinal ganglia at C5/C6 occurs in brachioradial pruritus (BRP) and causes unilateral or bilateral pruritus in the forearms [4].

Chronic compression of the dorsal root ganglion (CCD) is a preclinical model of lumbar intraforaminal stenosis and radicular pain [5, 6]. CCD increases the incidence of paw-shaking to a normally suprathreshold nociceptive force (“hyperalgesia”) [7, 8]. Hypersensitivity of mechanical behavior emerges in rats after CCD or after local application of proinflammatory tissue to the lumbar DRG [9–11]. The cell bodies of sensory neurons in the compressed DRG become hyperexcitable as evidenced by the presence of spontaneous activity originating in the DRG [12, 13], and elevated responses to electrical, thermal, and chemical nociceptive stimuli [14]. Although the pathophysiology of low back pain is well studied, the neural mechanisms accompanying itch are not largely explored.

Histamine is released from mast cells when tissues are inflamed or stimulated by allergens, and once released histamine-induced itch is triggered by the excitation of a subset of unmyelinated C-fibers [15]. BAM8-22 is an agonist which can bind and activate hMrgX1, mMrgC11 and rMrgC receptors with nanomolar affinities [16–18]. The study of BAM8-22 is of particular interest not only because of its important role in pain transmission and modulation, but also for its highest metabolic stability and long duration of action compared to other Mrg neuropeptides agonists [18].

The present study was designed to explore whether there might be an enhanced behavioral response to pruritogens in CCD mice and the potential role of primary pruriceptive neurons in mediating itch-related behaviors.

## Methods

### Animals

C57BL/6 male mice were used in all experiments. Male GCaMP 3^+^ mice and MrgprA3^+^ mice were used in confocal image experiments (Charles River, Wilmington, MA), each weighing 25–30 g and maintained on a 12-h light/dark cycle. The MRGPRA3 transgenic mice was provided by Dr Xinzhong Dong’s laboratory (John Hopkins University). For MrgprA3 + mice, the GFP was in DRG neurons that expressed the MRGPRA3 receptor. Mrgpra3 + neurons specifically responded to bam8-22 stimulation [19]. All animal welfare and experimental procedures were in strict accordance with the Guide for the Care and Use of Laboratory Animals and related ethical regulations of IBMS PUMC, according to the guidelines provided by the International Association for the Study of Pain and National Institutes of Health. Mice were given an adlibitum access to a standard diet and water. Mice were divided into a control group and a CCD group.

### Surgical treatment

Under 3% isoflurane anesthesia, a midline incision was made along the back, and the intervertebral foramina of L3 and L4 were exposed after separating the paraspinal muscles from the mammillary process and the transverse process. CCD was induced by the insertion of an L-shaped stainless steel rod (0.3 mm diameter, each arm, 2 mm in length), into each foramen to compress DRGs [7].

The incision was closed in layers and topically treated with ointment containing an antibiotic (TriTop), which is a local anesthetic and an anti-inflammatory agent. A systemic antibacterial was also administered (Baytril, 10 mg/mL, i.m.).

After completion of all behavioral testing, mice were euthanized, and DRGs receiving CCD were microscopically examined to confirm rod placement, after removal of the epineurium and flushing with saline.

### Behavioralassay

Three separate groups of mice were given subcutaneous injection of capsaicin (0.1, 1, 10 μg/10 μL; n = 6, 8, 6), histamine (10, 20, 50 μg/10 μL; n = 5, 8, 5),and BAM8-22(0.1, 1, 10 μg/10 μL; n = 5, 8, 8), into the calf of hind leg respectively and subsequent behavior was recorded for 30 min using a high definition camera on pre-CCD day 1 and post-CCD days 1, 3, 5,and 7. According to present studies, the injection of capsaicin brings out nociceptive (painful) sensations which lead to licking toward the injection site in calf models while pruritic stimulus generally arouses biting behaviors. Hence, the cumulative durations of licking and biting the injection site were counted via video, and used as an assessment of chemical-induced pain and itch, respectively.

The chamber was specially made from a cylindrical glass container (20 cm, diameter) with two small mirrors attached to plastic bricks placed as a right angle inside, offering a wide view of every act of the animal. There were 10 min of habituation before each test and recording started immediately after the injection.

### DRG exposure surgery for in vivo imaging of the whole L4 DRG

For all imaging experiments, mice 8 weeks or older were anesthetized by injection of sodium pentobarbital (40–50 mg/kg, i.p.). After deep anesthesia was reached, the animal’s back was shaved and aseptically prepared, and ophthalmic ointment was applied to the eyes to prevent drying. During the surgery, mice were kept on a heating pad (DC temperature controller, FHC) to maintain body temperature at 37 ± 0.5 °C as monitored by a rectal probe.

Dorsal laminectomy of the DRG was performed at the spinal level L5 to L3below the lumbar enlargement but without removing the dura. A 1.5 cm long midline incision was made around the lower part of the lumbar enlargement area, and these were dissected away to expose the lower lumbar part surrounding (L3–L5) vertebra bones. The L4 DRG transverse processes were exposed and cleaned. Using small rongeurs, the surface aspect of the L4 DRG transverse process near the vertebra was removed (only the L4 DRG transverse process was removed but the bone over the spinal cord was intact) to expose the underlying DRG without damaging the DRG and spinal cord. Bleeding from the bone was stopped using styptic cotton.

### In vivo L4 DRG calcium imaging

In vivo imaging of whole L4 DRG in live mice was performed for 5 days after CCD surgery. After surgery, mice were placed in a prone position on a designed microscope stage. The spinal column was stabilized using clamps to minimize movements caused by breathing and heart beats. Mice were maintained under continuous anesthesia for the duration of the imaging experiment with 1–2% isoflurane gas using a gas vaporizer. Pure oxygen was used to deliver the gas to the mouse.

The microscope stage was fixed under a laser-scanning confocal microscope (Nikon C2 microscope system), which was equipped with a macro based large objective and fast EM-CCD camera. Live images were acquired for 8–10 frames at600 Hz in frame-scan mode for 6–7 s, using a 5*0.5 N.A. macro dry objective, at a512*512 pixel resolution with solid diode lasers (Nikon) tuned at a 448 nm wavelength, and emission measured at 500–550 nm for green fluorescence. For analysis, raw image stacks (512*512 pixels in the x–y plane; approximately 8 optical sections) were imported into a Nikon Instrument system-element for further analysis. DRG neurons were positioned at the focal plane and imaging was monitored during activation of DRG neuron cell bodies by peripheral chemical stimuli. Imaging parameters were chosen to allow repeated imaging of the same cell over many stimuli, without causing damage to the imaged cells or to surrounding tissue.

### Calcium imaging measurement

Calcium imaging was conducted using a previously reportedexperimental method [20]. Briefly, we first chose neurons that respond to chemical stimuli as a region of interest (ROI). Then, we defined F0 as the average pixel intensity during the first 2–6 frames of every experiment. We then defined Ft as the maximum fluorescence intensity after chemical stimulation. We used a formula ΔF/F = (Ft–F0)/F0 to express the neuronal response to chemical stimuli.Wedefines calcium transients as chemically induced if they occur between the beginning of the chemical injection and up to 7 s after the end of the injection. A ΔF/F ratio greater than 0.8 was taken to indicate a positive neuronal response to the compound. Finally, the total number of reactive neurons was counted.

### Confocal imaging of DRGMrgprA3 + neurons in vitro

Six male MrgprA3 mice were selected to make CCD model. On the fifth day after operation, laminectomy was performed. The intact L4 DRG was removed by ophthalmic forceps and placed in a culture dish containing 37°Cartificial cerebrospinal fluid (ACSF). The whole DRG was scanned and photographed by Nikon stereoscopic confocal microscope. Neurons positive for MrgprA3 were excited at 448 wavelength, and emission measured at 500–550 nm for green fluorescence.

### Immunofluorescence

Five days after CCD surgery, the L3 and L4 DRGs of five mice were removed after transcardial perfusion with PBS followed by4% paraformaldehyde, and post-fixed in the same fixative for 4 h, before overnight cryoprotection in 30% sucrose. Tissue was frozen and sectioned ata12 μm thickness by cryostat and processed for immunofluorescence labeling [21]. The sections were dried at 37 °C for 1 h and fixed with 4% paraformaldehyde at room temperature for 10 min. The slides were preincubated in blocking solution (10% normal horse serum (vol/vol), 0.2% Triton X-100 (vol/vol) in PBS, pH 7.4) for 1 hr at room temperature, then incubated overnight at 4 °C with primary antibodies. Secondary antibody incubation was performed at room temperature for 1 h.

For primary antibodies, we used rabbit anti-CGRP (T-4239, Peninsula, 1:1,000), rabbit anti-HRH1 (13413-1-AP, 1:400), and guinea pig anti-TrpV1 (Abcam, 1:400).

For secondary antibodies, we used donkey anti-rabbit (A11008, Alexa 488 conjugated; A11011, Alexa 568 conjugated, Thermo Fisher), donkey anti-guinea pig (706-545-148, Alexa Fluor 680 conjugated; 706-625-148, Alexa Fluor 680 conjugated, Jackson lab). All secondary antibodies were diluted 1:500 in blocking solution. Following washes with PBS, the stained sections were mounted and cover-slipped with VECTASHIELD Mounting Medium (Vector Laboratories, Burlingame, CA, USA). The sections were examined and immunostaining images were obtained with an Olympus microscope.

For the analysis of immunohistochemical images, neurons exhibiting expression of at least one protein of interest (TRPV1 or/and H1R) were taken under consideration and analysed in a total of 12–16 randomly selected sections per group (4–6 sections per animal, 3 animals per group).

### Quantitative realtime-RT-PCR

The mRNA levels of TRPV1, Histamine receptor 1, Histamine receptor 4, and MrgprA3 receptor in the DRG were measured by real-time PCR (RT-qPCR). Total RNA was extracted using Trizol reagent according to the manufacturer’s instructions. The cDNA was synthesized from 1 µg of total RNA by the PrimeScriptTMRT reagent Kit with gDNA Eraser (Perfect Real Time). Each cDNA sample was amplified for the gene of interest and GAPDH in a 25 µL reaction volume using SYBR1 Premix Ex TaqTM II (TliRNaseH Plus). All primers used are listed in Table 1. The realtime RT-PCR conditions were 94 °C for 30 s followed by 40 cycles of 95 °C for 5 s, 55 °C for 30 s, and 72 °C for 60 s. The mRNA levels of all genes were normalized to GAPDH.

**Table 1:** The sequence of the primers used in the experiment

Gene	Primer sequence (5′-3′)
Trpv1-F	CCGGCTTTTTGGGAAGGGT
Trpv1-R	GAGACAGGTAGGTCCATCCAC
Hrh1-F	CAAGATGTGTGAGGGGAACAG
Hrh1-R	CTACCGACAGGCTGACAATGT
Hrh4-F	GTCCCCTTGGCATTTTTAATGTC
Hrh4-R	ACATGCAGATTCCACTTCCAAA
Mrgpra3-F	CTCAAGTTTACCCTACCCAAAGG
Mrgpra3-R	CCGCAGAAATAACCATCCAGAA

### Statistical analyses

For in vivo experiments, the animals were randomly distributed into various treatment groups. All of the results are given as means ± SEM. After determining the normality of the data, a one-way repeated-measures analysis of variance will be used for the comparison of data with normal distribution and the Kruskal–Wallis test will be used for data with non-normal distribution. A p < 0.05 will be considered indicative of statistical significance. When ANOVA analyses showed significant differences, pairwise comparisons between means were tested by the post hoc Tukey method (SigmaStat, San Jose, CA).

## Results

### Sensitized DRG neurons show enhanced response to chemical stimuli after CCD

**Fig. 1:**
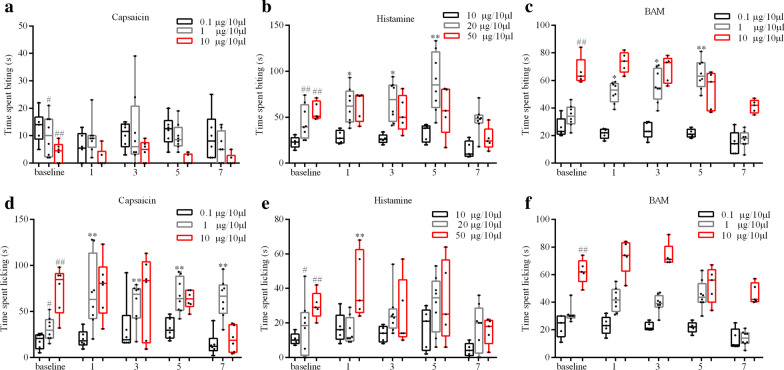
Capsaicin, Histamine, and BAM induces itch-like and pain-like behavior in the calf injection model

To explore the behavioral effects of TRPV1, Histamine, and MrgprA3 receptor activation after CCD, we used a calf model that allows differentiation of side-directed itch- and pain-like behaviors in response to pruritic and algesic chemical stimuli. Mice with a high dose of capsaicin displayed less site-directed biting behaviors than mice with a low dose (P < 0.05, 1 vs 0.1 μg/10 μL; P < 0.01, 10 vs 0.1 μg/10 μL) [Fig. 1a]. Mice with a high dose of capsaicin displayed more site-directed licking behaviors than mice withalow dose (P < 0.05, 1 vs 0.1 μg/10 μL; P < 0.01, 10 vs 0.1 μg/10 μL) [Fig. 1d]. Intradermal (i.d.) injection of capsaicin (1 μg/10 μL) into the calf of CCD mice significantly increased the number of licking but not bitingbouts as compared to mice before CCD (Fig. 1d). These results indicate that CCD caused a sensitization of capsaicin-evoked pain, but not cause a sensitization of capsaicin-evoked itch..

5 days after CCD surgery, mice injected with histamine displayed more site-directed biting behaviors than before CCD [Fig. 1b]. Mice withahigh histamine dose displayed more site-directed biting behaviors than mice receiving a low dose (P < 0.01, 20 and 50 μg/10 μL vs 10 μg/10 μL) [Fig. 1b]. Intradermal (i.d.) injection of histamine (20 μg/10 μL) in CCD mice significantly increased the number of biting but not licking bouts compared to pre-CCD (Fig. 1b, e). These results suggest that CCD caused a sensitization of histamine-evoked itch, but not cause a sensitization of histamine-evoked pain.. Future studies are needed to determine if an increase of animal sample sizes could detect a moderate change.Wenexttested BAM8-22, a histamine-independent pruritogen, on evoking skin itch. 5 days after CCD surgery, mice with BAM injection displayed more site-directed biting behaviors than before CCD [Fig. 1c]. Intradermal (i.d.) BAM injection (1 μg/10 μL) into the calf of CCD mice significantly increased the number of biting but not licking bouts as compared to pre-CCD (Fig. 1c, f). Intradermal (i.d.) BAM injection (10 μg/10 μL) in control mice significantly increased the number of licking bouts compared to BAM injection(1 μg/10 μL) [Fig. 1f]. These results suggest that CCD caused a sensitization of BAM-evoked itch, but not cause a sensitization of BAM-evoked pain.

To compare DRG neuron responses to chemical stimuli between pre-CCD surgery and 5 days after surgery, we found the most suitable chemical concentration. In low and high concentrations, there were no significantly different behavioral results between pre-surgeryand 5 days after CCD surgery.

### Confocal DRG imaging

To evaluate neuronal activity in DRG somata, we used Pirt-GCaMP3 mice to image the Ca^2+^ response to chemical stimuliin L4 DRG.

**Fig. 2:**
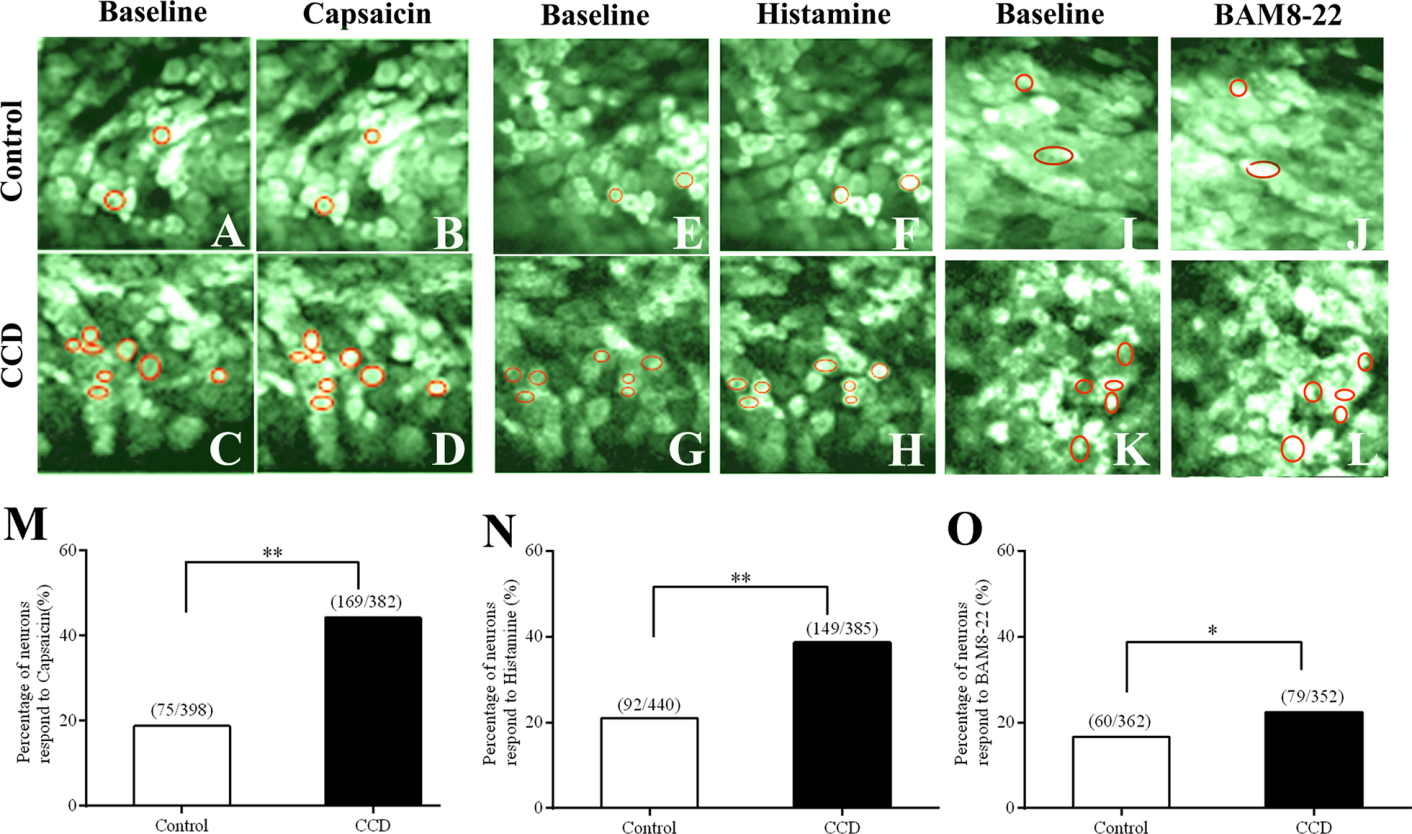
Calcium imaging of activity in L4 DRG neurons after CCD in pirt-GCaMP3s mice

The percentage of neurons that responded to capsaicin (1 µg/10 µL) was significantly increased in CCD (169/382, n = 6) compared with control DRG neurons (75/398, n = 4), P < 0.01, as shown Fig. 2a–d, m. The percentage of neurons responding to histamine (20 µg/10 µL) was significantly increased in CCD (149/385, n = 4) compared with the control DRG (92/440, n = 5), P < 0.01, as shown Fig. 2e–h, n.

The percentage of neurons that responded to BAM8-22 (1 µg/10 µL) was significantly increased in CCD (79/352, n = 3) compared with control DRG neurons (60/362, n = 3), P < 0.01, as shown Fig. 2i–l, o.

**Fig. 3:**
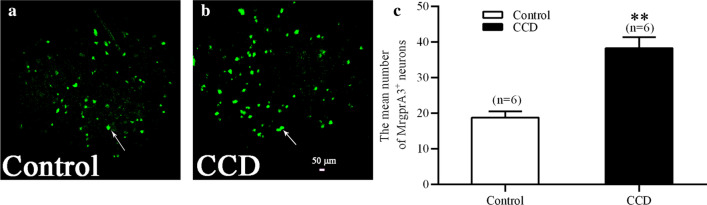
Upregulation of MrgprA3^+^ sensory neurons of L4 DRG in CCD mice

In the present study, a CCDmousemodel was used to mimic a chronic neuropathic state. Using confocal imaging, we investigated MrgprA3^+^ neuronal activity in L4 DRG somata. The mean number of MrgprA3^+^ neurons was significantly increased in CCD (sum = 230, 38.33 ± 3.07, n = 6) compared with the control DRG (sum = 113, 18.83 ± 1.7, n = 6), P < 0.01, as shown Fig. 3a–c.

### H1R and TRPV1 immunoreactivity inDRG neurons after CCD surgery

In our immunofluorescence staining study, immunoreactivity for TRPV1 and H1R in DRG neurons of CCD mice increased compared with control mice.

It is possible that DRGsensitization we detected after CCD is mediated by upregulated and spontaneously released histaminevia activation of HR1A on DRG neurons.

**Fig. 4:**
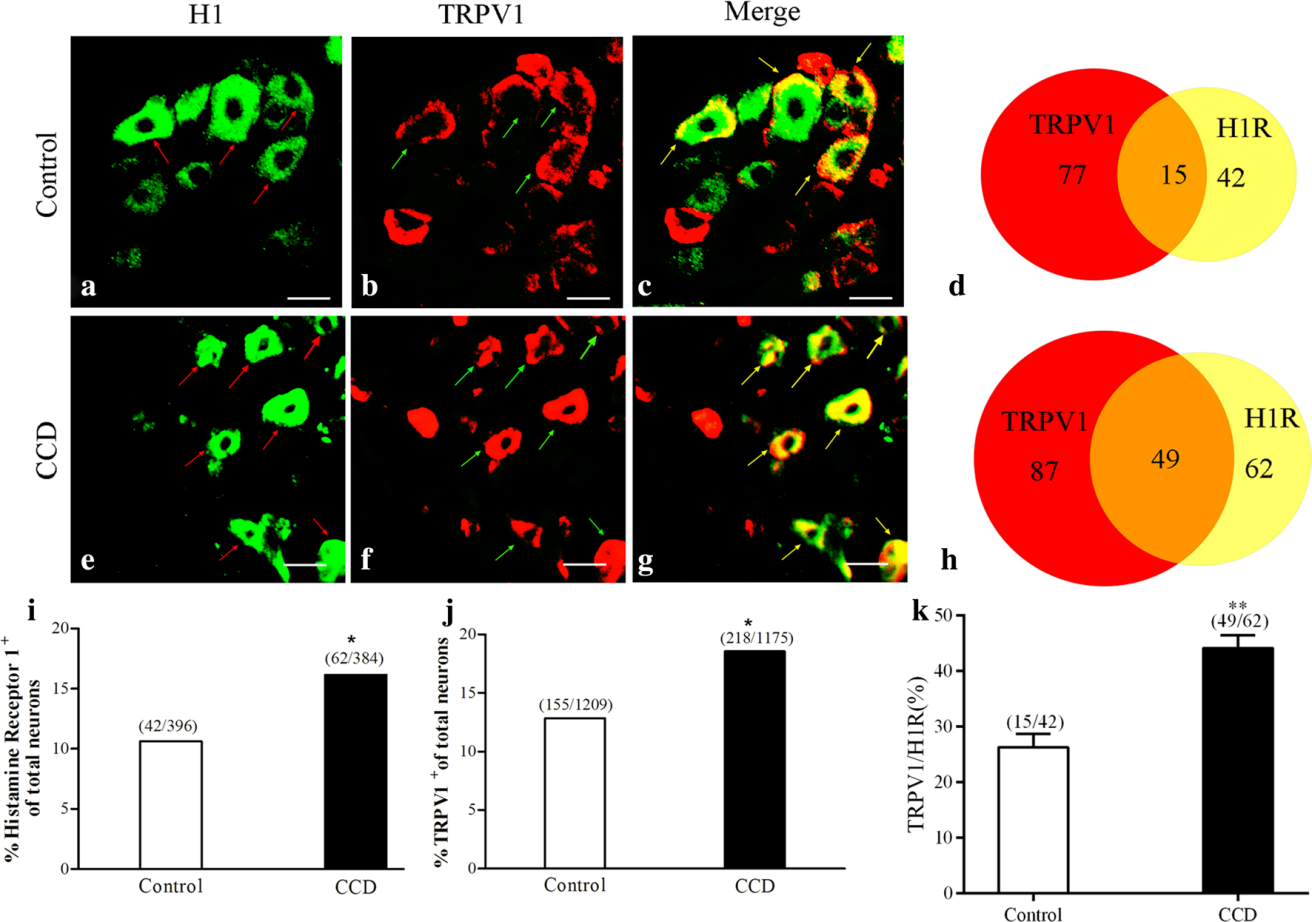
Immunofluorometric analysis of H1 and TRPV1 expressions in DRG neurons in CCD mice

Immunofluorescent staining revealed very few H1R- and TRPV1-immunopositive DRG neurons in control mice (Fig. 4a–c). In contrast, the mean percentage of H1R- and TRPV1-immunopositive DRG neurons was significantly greater in CCD mice (Fig. 4e–g). In addition, some H1R-immunopositive DRG neurons in CCD mice also showed significantly increased TRPV1 immunopositivity(detected in 44.14% of H1R-immunopositive neurons, Fig. 4h) compared with control mice (detected in 26.31% of H1R-immunopositive neurons, Fig. 4d). For H1R-positive neurons, in the control animals, small amount (26.31 ± 2.37%, 15/42) expressed TRPV1; in the CCDmice, the percentage were 44.14 ± 2.32% (49/62) at day 5 after CCD (p < 0.001) (Fig. 4k).

At the protein level, a significantly larger percentage of DRG neurons in CCD mice showedTRPV1immunopositivitycompared with controls (12.82%:155/1209, n = 10; Fig. 4i), indicating an increased number of neurons expressing TRPV1 after the development of CCD (18.55%:218/1175, n = 6).

We further determined the H1R expression pattern inDRG after CCD. H1R immunoreactivity was detected in 10.60% (42/396, n = 6) of DRG sensory neurons in controls. However, H1R immunoreactivity was detected in 16.15% (62/384, n = 6) of DRG sensory neurons in CCD mice, as shown in Fig. 4j.

**Fig. 5:**
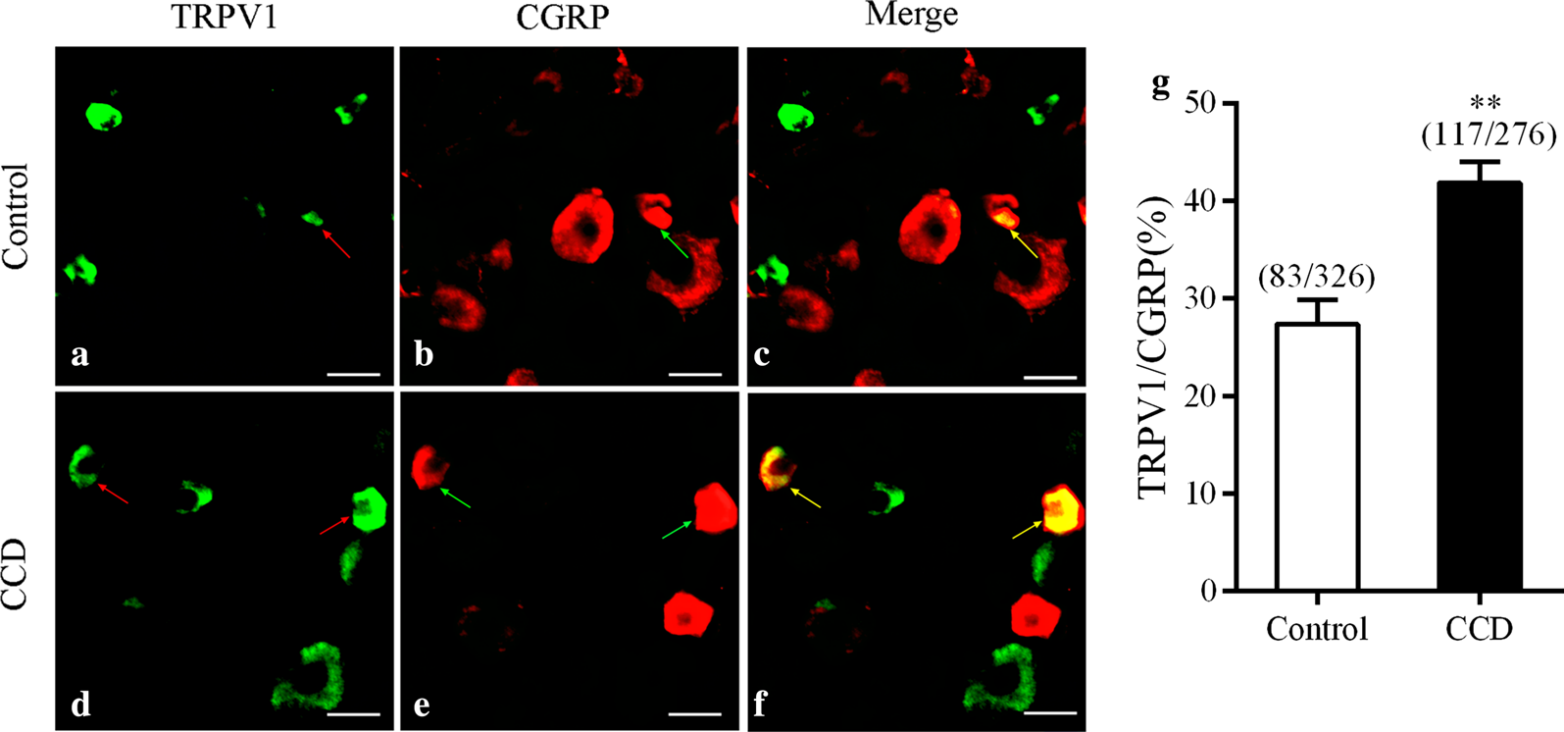
Immunofluorometric analysis of TRPV1 and CGRP expressions in DRG neurons in CCD mice

TRPV1^+^ DRG sensory neurons were generally small diameter neurons (average 30 µm), positive for CGRP. Immunofluorescent staining revealed very few TRPV1- and CGRP-immunopositive DRG neurons in control mice (Fig. 5a–c). In contrast, the mean percentage of TRPV1- and CGRP-immunopositive DRG neurons was significantly greater in CCD mice (Fig. 5d–f). For CGRP-positive peptidergic neurons, in the control animals, small amount (27.31 ± 2.54%, 83/326) expressed TRPV1; in the CCDmice, the percentage were 41.8 ± 2.22% (117/276) at day 5 after CCD (p < 0.001).

(Fig. 5g).

### CCD elevated TRPV1 and MrgprA3mRNA expression in DRG

Peripheral tissue and nerve injury can lead to hyperalgesia, a state in which painful or itching stimuli are perceived as more painful or itchy than normal.

**Fig. 6:**
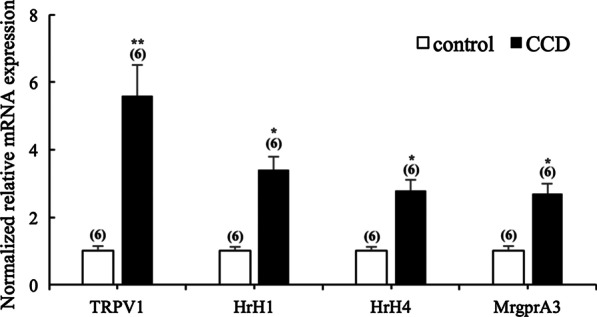
Upregulation of mRNA expression of TRPV1, HrH1, HrH4 and MrgprA3 in the DRG in CCD mice

Pathological conditions such as inflammation and nerve injury can sensitize DRG neurons, causing heightened pain sensitivity and often leading to chronic pain. To further examine the contribution of TRPV1, histamine receptor 1 and MrgprA3 in DRG primary sensory neurons to persistent pain and itch after CCD, we tested mRNA expression of the three receptors in control and CCD DRG neurons. As shown in Fig. 6, mean mRNA expression levels of TRPV1, histaminereceptor 1 and MrgprA3 were significantly elevated in the CCD group compared to control group (P < 0.05).

## Discussion

In this study, we found that compressed DRG neurons showed significantly enhanced responses to histamine and BAM8-22.

Previous research found that H1R and H4R are both expressed on C-afferent fiber terminals, and that these antagonists can directly inhibit the transmission of itching responses from the peripheral to central nervous system [22]. Using single-cell calcium imaging, Rossbach et al. found that histamine induced an increase in calcium levels in a subset of skin-specific sensory neurons in mice by activating H1R and H4R, and inhibiting H3R [23].

Histamine is a well-known mediator of acute inflammatory and immediate hypersensitivity responses. Though the physiological role of histamine is well studied, less is known of the signaling pathway leading to excitation of sensory neurons, which induces the adaptation of neural signals for itching. Our study provides in vivo and in vitro evidence supporting that histamine requires TRPV1activation to excite DRG primary sensory neurons after CCD. H1R can activate phospholipase C, and increases intracellular Ca^2+^levels. Pruritus is elicited by the activation of H1R.

The transient receptor potential cation channel, subfamily V, member 1 (TRPV1) is an important molecular component of pain detection and modulation at peripheral and central nociceptive neurons [24, 25]. Moreover, research showed that histamine induces itch by activating PLA2, lipoxygenase, and the TRPV1 signaling pathway [26]. Histamine induces inward currents that are blocked byTRPV1antagonists [26, 27].

The strong relationship between histamine and TRPV1 in DRG primary sensory neurons was shown in our study. Coexpression of TRPV1 and histamine receptors occurs in a subset of sensory neurons [28, 29], and primary afferent C-fibers that respond to histamine are also sensitive to capsaicin [28, 30]. Moreover, repetitive capsaicin application is known to desensitize TRPV1 or sensory nerves and also to alleviate pruritus induced by histamine [31]. These combined results further strengthen the notion that TRPV1 mediates histamine-induced itching. There are reports suggesting that histamine H1 and H4 receptors are co-involved in the pathway to transmit the itch signal to the central system [32, 33]. In the present study, we showed that CCD increased H1R and TRPV1 agonist-induced itching behaviors.After H1 receptor activation, the Gαq proteins, coupled with the H1 receptor downstream signal pathway, induced TRPV1 to open and excite the neurons to transmit the itch signal [26, 27].

The neurons activated by histamine at high concentration are not the same group of neurons that activated by histamine at low concentration. According to the results of animal behavior experiments, we found that there was no significant difference in the behavior of pain and itching between normal mice and CCD mice after low concentration of histamine. Similarly, there was no statistically significant difference in pain and itching behavior between the two groups of mice injected with high concentrations of histamine. Injection of BAM and capsaicin had the same situation.

Han’s study found that MrgprA3 + neurons are also TRPV1 + and sensitive to capsaicin [19]. MrgprA3 neurons had cutaneous nociceptors with C-fibers that responded to the chemicals (histamine, BAM8-22 and capsaicin) were injected intradermally into the receptive field. Selective activation of the pruriceptive neuron by an injection of capsaicin in a mouse in which the capsaicin receptors has been knocked out.

BAM8-22, a fragment from the proenkephalin A gene, is a ligand capable of potently activating rat MrgC11 and MrgA3. After CCD, the expression of MrgA3 protein in DRGwaselevated compared to control mice.

In this study, mRNA inDRG tissue was investigated. Future studies should focus on single cell PCR to detect neuronal mRNA expression of histamine receptors and TRPV1. Undoubtedly, more work is needed to understand how these pruriceptors enhance pruriceptic behaviors in mouse.

## Conclusions

In summary, our study showed that lower concentrations of histamine and BAM8-22 excited sensory neurons to induce more itching behavior in the CCD mice compare with control mice.

The responses of compressed DRG neurons to histamine and BAM8-22 were significantly enhanced, and H1R and TRPV1in CCD DRG neurons were markedly upregulated. Considering that histamine is an important cause of itching in dermatitis patients, our study provides clues concerning the treatment of evoke ditching and inflammatory pain.

## Data Availability

There is no data, software, databases, and application/tool available apart from the reported in the present study. All data is provided in manuscript and supplementary data. All authors read and approved the final manuscript.

## Abbreviations

CCD: Chronic compression of dorsal root ganglion
DRG: Dorsal root ganglion
CGRP: Calcitonin gene-related peptide
TRPV1: Transient receptor potential vanilloid-1
BAM: Bovine adrenalmedulla8-22
5-HT: 5-Hydroxytryptamine
H1R: Histamine receptor 1
